# The DNA methylome of inflammatory bowel disease (IBD) reflects intrinsic and extrinsic factors in intestinal mucosal cells

**DOI:** 10.1080/15592294.2020.1748916

**Published:** 2020-04-12

**Authors:** Iolanda Agliata, Nora Fernandez-Jimenez, Chloe Goldsmith, Julien C. Marie, Jose R. Bilbao, Robert Dante, Hector Hernandez-Vargas

**Affiliations:** a Department of Medicine and Health Sciences, University of Molise, Campobasso, Italy; b Department of Genetics, Physical Anthropology and Animal Physiology, University of the Basque Country (UPV/EHU) and Biocruces-Bizkaia Health Research Institute, Leioa, Spain; c Department of Immunity, Virus and Inflammation, Cancer Research Centre of Lyon (CRCL), Inserm U 1052, CNRS UMR 5286, Université de Lyon, Centre Léon Bérard, Lyon, France; d Ciber de Diabetes y Enfermedades Metabólicas Asociadas (CIBERDEM), Madrid, Spain; e Department of Signaling of Tumoral Escape, Cancer Research Centre of Lyon (CRCL), Inserm U 1052, CNRS UMR 5286, Université de Lyon, Lyon, France; f Department of Translational Research and Innovation, Centre Léon Bérard, Lyon, France

**Keywords:** DNA methylation, inflammatory bowel disease (IBD), biomarkers, coeliac disease (CeD), methylation quantitative trait loci (mQTLs)

## Abstract

Abnormal DNA methylation has been described in human inflammatory conditions of the gastrointestinal tract, such as inflammatory bowel disease (IBD). As other complex diseases, IBD results from the balance between genetic predisposition and environmental exposures. As such, DNA methylation may be the consequence (and potential effector) of both, genetic susceptibility variants and/or environmental signals such as cytokine exposure. We attempted to discern between these two non-excluding possibilities by performing a combined analysis of published DNA methylation data in intestinal mucosal cells of IBD and control samples. We identified abnormal DNA methylation at different levels: deviation from mean methylation signals at site and region levels, and differential variability. A fraction of such changes is associated with genetic polymorphisms linked to IBD susceptibility. In addition, by comparing with another intestinal inflammatory condition (i.e., coeliac disease) we propose that aberrant DNA methylation can also be the result of unspecific processes such as chronic inflammation. Our characterization suggests that IBD methylomes combine intrinsic and extrinsic responses in intestinal mucosal cells, and could point to knowledge-based biomarkers of IBD detection and progression.

## Graphical abstract

**Figure:**
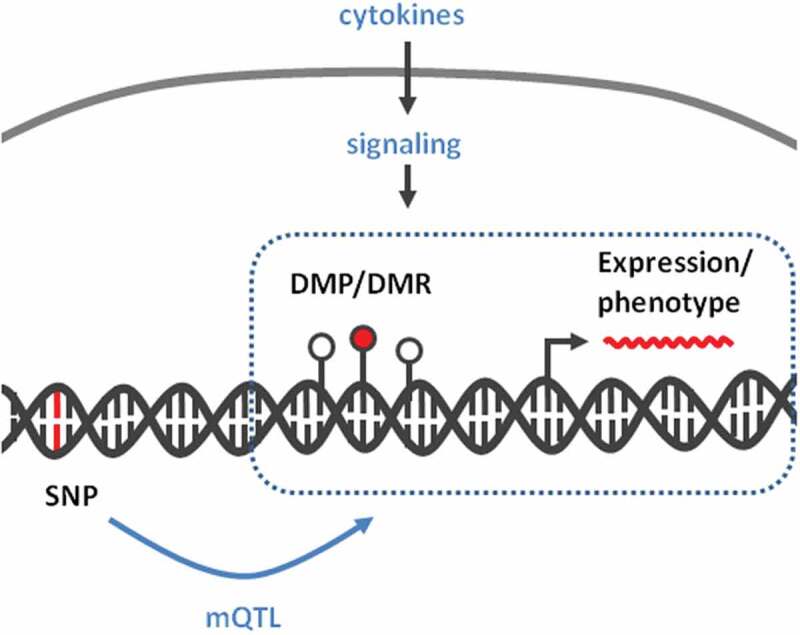

## Background

Inflammatory bowel disease (IBD) comprises Crohn’s disease (CD) and Ulcerative Colitis (UC), two chronic and progressive inflammatory conditions of the gastrointestinal (GI) tract that affect 2.2 million people in Europe and 1.4 million in United States [1,2]. The exact aetiology is not known, but IBD is characterized by various genetic abnormalities that result in aggressive response from both innate (i.e., macrophages and neutrophils) and acquired (i.e., T and B cells) immunity [3]. In CD, although inflammation may involve the entire GI tract, the ileum is mainly affected [4]. In UC, chronic and relapsing inflammation affects the colon and rectum [5] and is associated with increased risk of colon cancer development [6].

While genetics explains a fraction of inheritance of IBD (13,1% variance in CD and 8,2% in UC) [7], environmental factors may influence susceptibility through non-genetic mechanisms, such as DNA methylation [8,9]. Indeed, several recent studies have provided a detailed characterization of genomic abnormalities in IBD, including DNA methylation [10–12]. Although there is a clear crosstalk between DNA methylation and gene expression, the cause–effect relationship between these two processes is dependent on the biological context [9,13]. There is evidence for gene expression preceding DNA methylation changes [14–16], as well as evidence for DNA methylation as an effector of genetic variants and the resulting pathological phenotype [8]. Unifying both possibilities, DNA methylation may represent a mechanism to condition or to perpetuate the response to anti- and pro-inflammatory signals. For example, exposure to cytokines such as interleukin 6 (IL6) and transforming growth factor beta (TGF-β) has been associated with stable DNA methylation changes in epithelial cells [14,17–19]. However, it is unclear to what extent the altered DNA methylation of epithelial cells in IBD could be due to persistent cytokine exposure and/or to the direct consequence of genetic susceptibility variants (i.e., SNPs).

Explaining the origin of DNA methylation changes in IBD may be of interest when exploiting their potential as biomarkers. Currently, the most used biomarkers for IBD are C-Reactive Protein and Calprotectin, although they are not specific for inflammation of intestinal origin, limiting their clinical use [20]. Instead, DNA methylation is known to be tissue-specific [21,22], and it may represent a sensor of cytokine exposures [23–26] and thus a better biomarker of IBD. Moreover, DNA markers are advantageous in terms of stability, improved isolation and storage, relative to RNA or protein [27]. With these assumptions, we performed a combined analysis of intestinal epithelium methylomes in IBD. Our goal was to identify candidate loci that can be potentially useful as biomarkers, using base-resolution methylation data in mucosal biopsies from a large aggregated dataset of CD and UC patients, an approach that may open the way to personalized prevention strategies.

## Results

### Genome-wide changes in DNA methylation are a common feature of IBD

To identify DNA methylation changes in cells of the intestinal mucosa associated with IBD, we reanalysed bead-array methylation data from different datasets (Tables 1 & 2). To increase coverage while enhancing data harmonization, we only included datasets based on the last two versions of Illumina methylation bead arrays (i.e., HM450 and EPIC, see Methods for other inclusion criteria) which share ~400 k informative features. Samples from these datasets included paediatric and adult IBD patients, from both sexes, and involved the two main forms of the condition (i.e., CD and UC).

**Table 1.** **D**ataset characteristics.

Accession	Condition	idat	Samples	Age	Origin	PMID
MTAB_5463	UC/CD	yes	111/104	6-15	Europe	29031501
GSE32146	UC/CD	no	25	14-17	USA	NA
MTAB_3703/3709	UC/CD	yes	12	12-14	Europe	2376367
GSE81211	UC	yes	12	16-68	South Korea	27517910
GSE105798	CD	yes	11	NA	South Korea	NA
GSE42921	UC/CD	no	23	5-19	USA	NA

Characteristics of the datasets included in the study. Accession: either ArrayExpress or Gene Expression Omnibus (GEO) accession numbers. Condition: ulcerative colitis (UC) or Crohn’s disease (CD). PMID: PubMed ID. Idat: raw-level bead-array data availability. NA: not available.

**Table 2.** Samples used in the study.

Accession	Array	Controls	Cases	Protocol	Segment	Inflammation
MTAB_5463	EPIC	20	84	IEC purification by sorting	sig: 53, ter: 51	Histology score available^a^
MTAB_5463	HM450	33	78	IEC purification by sorting	sig: 48, ter:48, asc: 15	Histology score available^a^
GSE32146	HM450	10	15	Intestinal mucosa	Colon	NA
GSE81211	HM450	3	8	Intestinal mucosa	Rectum	Endoscopic score available^b^
GSE105798	HM450	3	8	Intestinal mucosa	NA	NA
GSE42921	HM450	12	11	Intestinal mucosa	Colon	NA

Characteristics of the samples included in each study. Accession: either ArrayExpress or Gene Expression Omnibus (GEO) accession numbers. Array: Illumina bead array version. ^a^inflammation score available for each sample. ^b^active vs. inactive status, not attributable to each sample. NA: not available. In Segment, sig: sigmoid colon, ter: terminal ileum, asc: ascendent colon.

**Figure 1.**
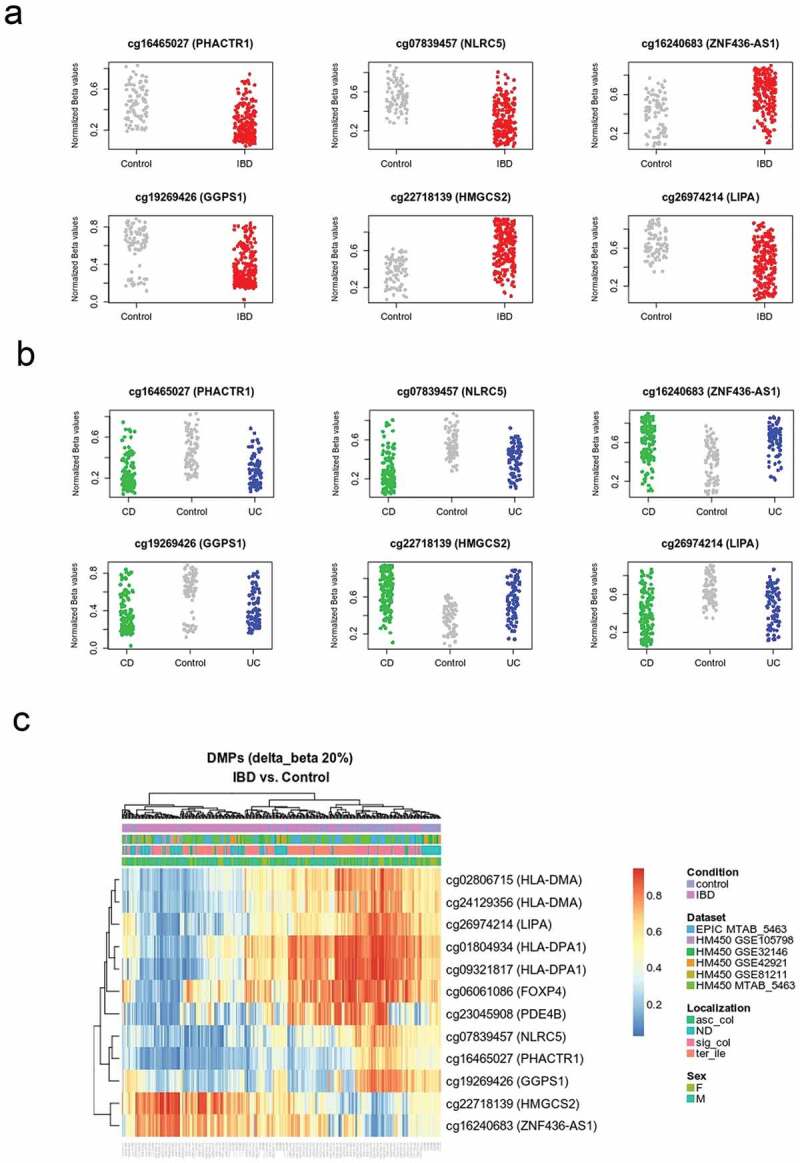
DNA methylation distinguishes IBD from healthy intestinal epithelial cells.

After filtering (see Methods), we tested for the association between IBD and DNA methylation at 392810 CpG sites (81 control and 204 IBD patients) using a linear model. In such a model, we adjusted for sex, age, dataset, and surrogate variables identified during data preprocessing (Figure S1). To account for statistical inflation, we used criteria of effect size (change in mean methylation of at least 10% between controls and IBD) and FDR-adjusted p value <0.05. Using these criteria, we identified 4205 differentially methylated positions (DMPs), out of which 436 were hypo- and 3769 were hypermethylated in IBD (Figure 1(a), Tables 3 and S1). DMPs were robust to IBD type (Figure 1(b)), and other clinical and technical features (Figure 1(c), S2, and S3). An important fraction of these sites was previously identified, in particular in the large dataset published by Howell et al. [10]. However, our dataset combination strategy has led to the identification of new associations. Moreover, the consistency of these findings across independent studies provides additional confidence on their robustness.

**Table 3.** Top DMPs.

Probe ID	FDR	Delta Beta	Symbol	Distance
cg16465027	1.14E-14	-0.20	*PHACTR1*	122016
cg19269426	1.80E-12	-0.22	*GGPS1*	0
cg07839457	6.58E-12	-0.23	*NLRC5*	435
cg16240683	1.24E-10	0.22	*ZNF436-AS1*	0
cg24129356	4.02E-09	-0.22	*HLA-DMA*	0
cg22718139	1.05E-08	0.25	*HMGCS2*	0
cg26974214	5.56E-08	-0.23	*LIPA*	0
cg02806715	3.03E-07	-0.20	*HLA-DMA*	0
cg09321817	7.33E-07	-0.25	*HLA-DPA1*	0
cg01804934	2.60E-05	-0.21	*HLA-DPA1*	0
cg23045908	6.30E-05	-0.21	*PDE4B*	0
cg06061086	9.70E-04	-0.20	*FOXP4*	14522

Differentially methylated positions (DMPs) with a mean difference between groups of at least 20% (delta-beta -CBrk- 20, FDR -OBrk- 0.05). Probe ID: Illumina probe reference, logFC: logarithmic fold-change between groups (IBD vs. control), FDR: false discovery rate, Symbol: gene symbol, Distance: distance in base pairs to the closest gene. Full list of DMPs can be found in Supplementary Table S1.

(a) Top differentially methylated positions (DMPs) with a mean difference between IBD (red) vs. Control (grey) of at least 20% (delta-beta > 20, FDR < 0.05). Probe ID and corresponding nearest gene are shown for each significant CpG site. Methylation is represented on the y-axis as normalized beta values. (b) The same CpG sites shown in (a) are represented separately for ulcerative colitis (UC) and Crohn’s disease (CD), shown in blue and green, respectively. (c) Heatmap showing top differentially methylated positions between IBD *vs.* control. The red to blue colour gradient represents higher to lower methylation. Main covariates considered in the analysis (i.e., dataset, anatomical location, and sex) are also represented.

**Figure 2.**
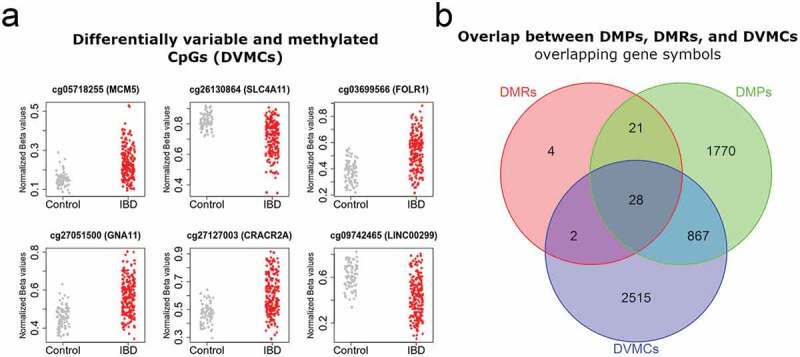
Mean DNA methylation and variability distinguishes IBD from healthy intestinal epithelial cells.

A subset of DMPs mapped close to each other, suggesting a non-random association with particular genomic loci. To explore this observation, we performed a region-level analysis in the same combined dataset. This led to the identification of 55 differentially methylated regions (DMRs), 31 hypo and 24 hyper methylated in IBD (Tables 4 and S2). As expected, many of these regions corresponded to gene loci also identified using the probe-level strategy (Figure 2(b)).

**Table 4.** Top DMRs.

Symbol	Coordinates	# CpG	FDR	beta FC
*BST2*	chr19:17516282-17517008	5	7.82E-60	-0.08
*HMGCS2*	chr1:120311439-120311653	4	1.37E-54	0.06
*HLA-DPA1*	chr6:33040535-33041697	8	5.01E-44	-0.07
*LGALS3*	chr14:55602634-55604454	4	1.14E-29	0.06
*LTBP3*	chr11:65318624-65318683	2	1.65E-36	0.05
*DACT2*	chr6:168665386-168665533	3	1.92E-31	0.05
*DYSF*	chr2:71823484-71823517	2	7.51E-29	0.05
*IARS2*	chr1:220292508-220292590	2	3.27E-27	0.06
*CIITA*	chr16:10969805-10971250	6	1.27E-37	-0.06
*VILL*	chr3:38033516-38033934	3	1.11E-29	0.06
*HLA-B*	chr6:31322298-31323856	5	3.04E-25	-0.05
*THBS1*	chr15:39874776-39876248	4	4.62E-16	0.07
*CLDN4*	chr7:73223852-73223935	2	5.85E-23	0.06
*CLHC1*	chr2:55360999-55361310	2	4.80E-23	-0.08
*ZNF467*	chr7:149462836-149463219	2	4.80E-24	0.05
*SHH*	chr7:155617333-155617398	2	1.30E-24	0.07
*ARL14*	chr3:160395420-160395719	2	1.65E-21	0.08
*TMEM232*	chr5:110062343-110062837	7	4.54E-22	0.07
*HOXA10*	chr7:27217057-27217606	2	1.09E-23	0.05
*FAAP20*	chr1:2120985-2121724	6	5.17E-21	0.07
*EPHB3*	chr3:184297380-184297522	3	5.82E-18	-0.09
*PSMD1*	chr2:231989800-231989824	2	7.41E-17	-0.08
*MUC4*	chr3:195552341-195552429	2	1.53E-16	-0.08
*SBNO2*	chr19:1130866-1130965	2	2.53E-18	-0.07
*PARP9*	chr3:122281881-122281975	3	2.17E-19	-0.08
*HOXD8*	chr2:176996285-176997707	5	5.07E-14	-0.05
*CUX1*	chr7:101579003-101579936	3	5.12E-11	0.05
*ATXN7L1*	chr7:105279391-105279882	3	4.08E-17	0.05
*ELL*	chr19:18589848-18589894	2	4.50E-14	-0.05
*PLA2G2A*	chr1:20305344-20305685	2	2.08E-13	-0.08
*SUMO1P1*	chr20:52356911-52357117	2	3.12E-13	-0.07
*TRIM69*	chr15:45018591-45018905	3	8.08E-21	-0.05
*HLA-C*	chr6:31238245-31238751	3	9.95E-16	-0.06
*SFT2D3*	chr2:128453108-128453484	5	7.96E-13	0.08
*STAT1*	chr2:191875807-191876673	2	1.99E-10	-0.08
*RPS6KA2*	chr6:166970252-166970727	2	3.48E-11	-0.05
*PHACTR1*	chr6:12594257-12595019	2	1.33E-21	-0.07
*SLC45A4*	chr8:142255356-142255537	2	1.54E-11	-0.06
*CANT1*	chr17:76991208-76991253	2	1.02E-14	0.05
*DRD3*	chr3:113933104-113933175	2	1.63E-09	-0.07
*AFF3*	chr2:100170766-100171136	4	1.54E-09	0.05
*LRRC47*	chr1:3720588-3720744	2	1.29E-09	0.06
*MIR34A*	chr1:9224003-9224198	2	2.40E-10	-0.06
*TRIM5*	chr11:5710654-5711068	2	7.64E-15	-0.06
*FOXP4*	chr6:41499640-41499799	2	7.46E-08	-0.05
*ATP9A*	chr20:50312386-50312632	2	1.01E-07	-0.05
*SLCO3A1*	chr15:92612836-92613280	2	8.59E-08	-0.05
*CCDC155*	chr19:49891494-49891574	2	3.13E-06	-0.06
*SVIL*	chr10:29948324-29948428	2	1.17E-05	-0.06
*TPM1*	chr15:63337352-63337496	2	8.85E-06	-0.05
*SHTN1*	chr10:118652838-118652981	2	1.18E-05	-0.06
*CES1P1*	chr16:55794595-55794731	2	3.09E-05	-0.06
*GNL3*	chr3:52722445-52722452	2	4.73E-05	0.06
*CYP2E1*	chr10:135341870-135342620	5	1.16E-05	0.05
*ASPG*	chr14:104552032-104552209	3	8.13E-05	0.05

Differentially methylated regions (DMRs) with at least five CpG sites, a maximum beta change between groups (beta FC) of at least 10%, and a minimum FDR of 0.05, are shown below. # CpGs: number of CpG sites per region, FDR: false discovery rate, Beta FC: methylation beta value fold change. Full list of DMRs can be found in Supplementary Table S2. The table is sorted by beta FC, from hypo- to hypermethylation in IBD.

(a) Top differentially variable methylated CpG sites (DVMCs) in IBD *vs.* Control. DNA methylation was plotted as beta values for each of the top nine DVMC identified with the iEVORA algorithm (see Methods section). (b) Gene symbols overlapping between site- (DMPs), region (DMRs)-level, and variability (DVMCs) analyses.

In addition, to mean methylation differences at the probe and region levels (i.e., DMPs and DMRs), methylation variation has been associated with disease and cancer susceptibility [28]. To explore this, we used the iEVORA algorithm in the same datasets, to identify differentially variable and methylated CpGs (DVMCs). Using stringent criteria of differential methylation and variation, we identified 4532 DVMCs (Figure 2(a) and Table S3), most of them located in the vicinity of a known promoter (80%, within 2 kb of a transcription start site). Of note, for most of these sites (75%), IBD samples displayed higher variability than control tissues. In addition, more than half of them displayed lower methylation in IBD samples relative to control mucosa (63%).

In summary, the intestinal mucosa of IBD displays large non-random methylome abnormalities characterized by high variability, but also by absolute changes in mean DNA methylation at particular loci.

### Genomic and biological context of IBD-associated DNA methylation changes in intestinal epithelia

**Figure 3.**
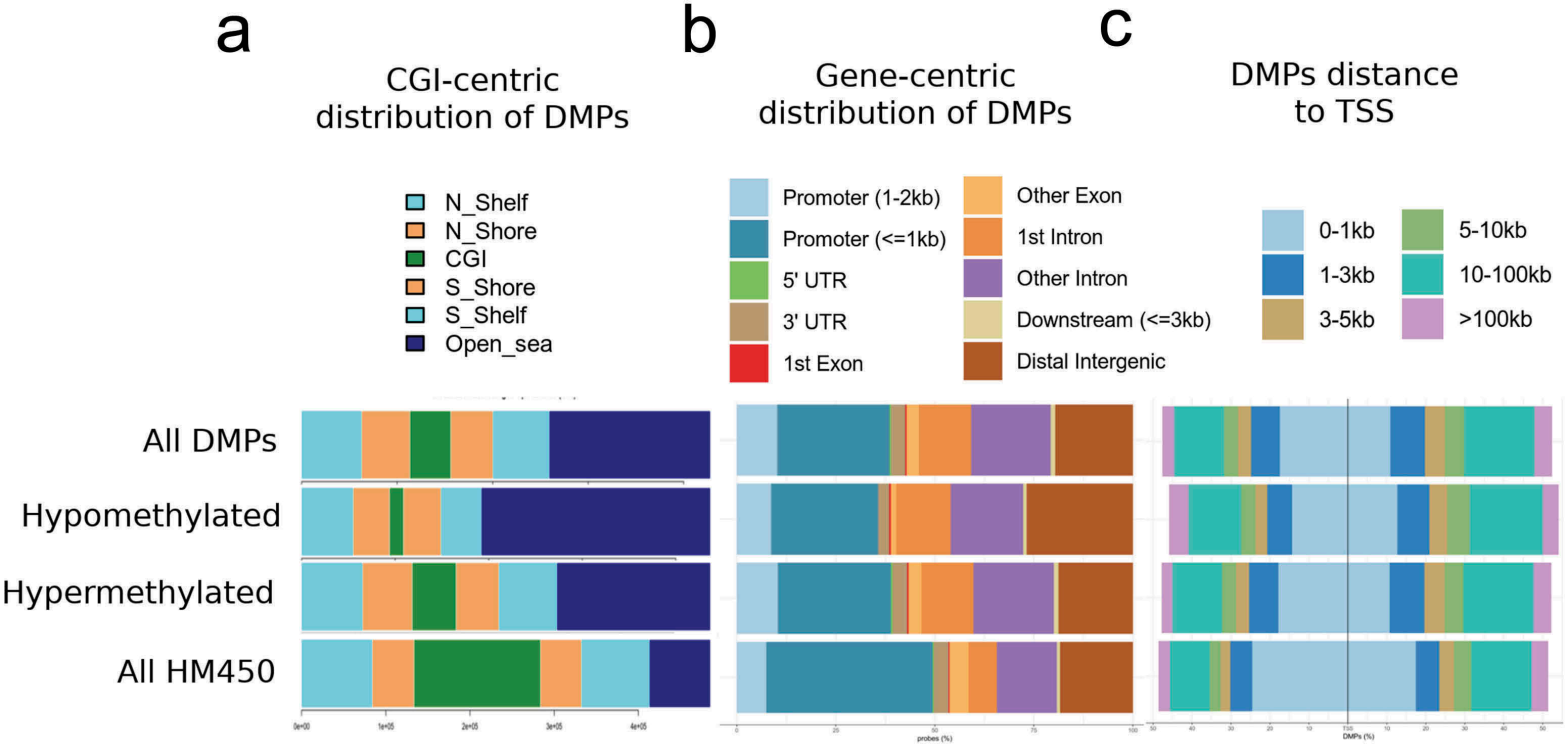
Genomic distribution of IBD-related DMPs.

DMPs distinguishing IBD from control tissues were assessed for genomic distribution, in terms of gene-centric and CpG island (CGI)-centric context. DMPs were relatively absent from CGIs, gene promoters, or the vicinity of transcription start sites (TSS) (Figure 3(a-c)). Instead, hypo and hypermethylated DMPs were highly concentrated in non-CGI regions (i.e., open sea) (Figure 3(a)). Pathway analysis of DMRs revealed over-representation of pathways related to metabolism and signal transduction, including Adipogenesis, Haemostasis, G alpha signalling events, Pathways in cancer, and TGF-beta Receptor Signalling (Table 5).

**Table 5.** Pathway analysis.

Pathway	Adjusted *P*-value	Combined Score	Dataset
*Adipogenesis genes_Mus musculus_WP447*	5.78E-06	33.17	WikiPathways 2016
*Adipogenesis genes_Homo sapiens_WP236*	5.78E-06	32.1	WikiPathways 2016
*TGF Beta Signalling Pathway_Mus musculus_WP113*	4.28E-04	26.34	WikiPathways 2016
*TGF-beta Receptor Signalling_Homo sapiens_WP560*	7.32E-04	24.45	WikiPathways 2016
*Alpha6-Beta4 Integrin Signalling Pathway_Mus musculus WP488*	2.44E-03	18.52	WikiPathways 2016
*Haemostasis_Homo sapiens_R-HSA-109582*	1.31E-03	29.15	Reactome
*G alpha(12/13) signalling events_Homo sapiens_R-HSA-416482*	4.29E-03	21.79	Reactome
*Pathways in cancer _Homo sapiens_hsa 05200*	5.90E-04	26.06	KEGG 2016
*Aldosterone synthesis and secretion_Homo sapiens_hsa04925*	5.90E-04	24.68	KEGG 2016

DMPs were annotated according to CpG islands (CGI) (a), relation to gene features (b), and distance to the nearest transcription start site (TSS) (c) For each genomic context, distribution is shown separately for all DMPs, those hypo- or hypermethylated in IBD relative to healthy tissues, and all the HM450 probes, as a control.

Overall, abnormal DNA methylation in IBD is relatively absent from CGIs. At the biological level, DNA methylation changes are enriched in inflammation-related pathways. Such changes may occur downstream of cytokine signalling. Alternatively, they may represent early changes linked to genetic susceptibility.

### IBD DMPs are genomically closer to IBD risk polymorphisms and are enriched on blood mQTLs

**Figure 4.**
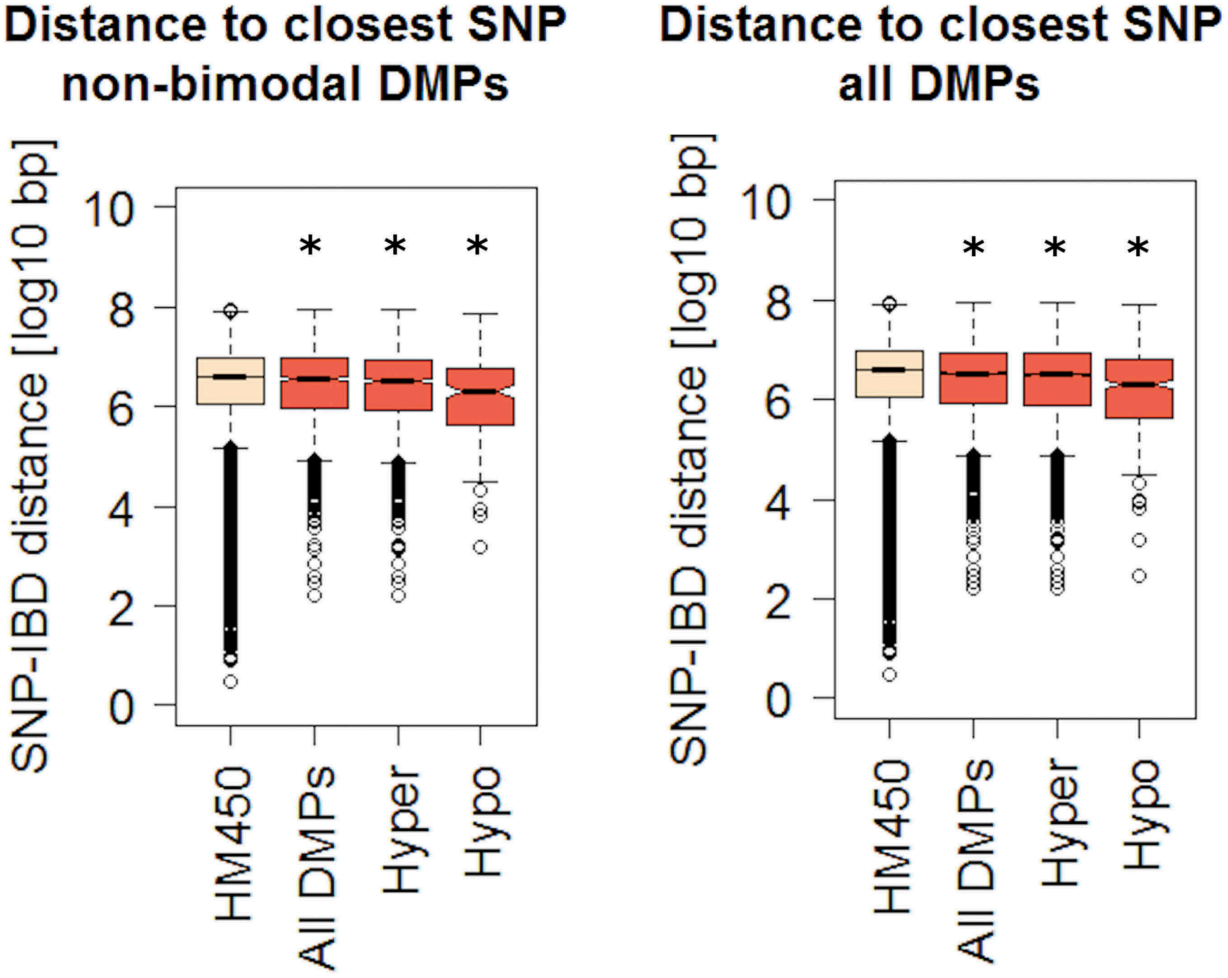
Genomic distances between IBD-related DMPs and known risk SNPs.

DNA methylation may represent an intermediary between genotype and disease susceptibility, and such genetic influences on DNA methylation within a defined genomic context are known as methylation quantitative trait loci (mQTLs). Among differentially methylated genes with a significant genetic association, we found *JAK3, KRT8*, and *HLA* genes, confirming the findings of previous studies [7,29–31]. Moreover, some DMPs display a bimodal DNA methylation distribution (see Methods). After ruling out technical artefacts, such bimodal distribution may suggest that their methylation levels are directly dependent on genotype. To explore a genotype-methylation association, we calculated the genomic distance between DMPs identified in our analysis and single nucleotide polymorphisms (SNPs) associated with IBD risk [29,30,32]. Of note, DMPs were overall significantly closer to a known IBD risk SNP, compared to all HM450 sites taken together (Figure 4). This difference was preserved after independently comparing hyper or hypomethylated DMPs (although more evident in the latter), and consistent across three independent SNP datasets (Figures 4 and S2C).

Shortest genomic distances were calculated between each IBD-related DMP and the closest IBD-associated polymorphism (SNP). Boxplots represent the distribution of such distances for all DMPs or separately for hyper- or hypo-methylated DMPs. The distance of all HM450 CpG sites was calculated as a control (left boxplot in both panels). The same analysis was performed for all DMPs (right panel) or using only DMPs that did not display a bimodal distribution (left panel), as described in Methods. (*) denotes a significant difference in mean distance relative to control HM450 distances (p < 1e-5).

We also tested the overlap between IBD-DMPs and CpGs participating in blood mQTLs as defined by McRae et al. [33]. Although this was not a significant enrichment, 544 out of the 4205 DMPs participated in the 52916 mQTLs reported previously (Supplementary Table S4). To ascertain whether the SNPs putatively associated to our DMPs were also associated to IBD, we interrogated the largest fine-mapping study performed to date on the disease that claims to identify associations at a base-pair resolution level [29]. We found that 4 of the 544 mQTLs identified here bear an IBD-associated polymorphism, namely rs11264305, rs17228058, rs3806308, and rs3807306, located in or close to *ADAM15, SMAD3, RNF186,* and *IRF5*, respectively. Briefly, we found that SNP-CpG pairs overlap regulatory loci, discernible by H3K27ac histone marks and the presence of a CpG island (in the case of *ADAM15*).

These findings suggest that at least a fraction of IBD abnormal methylome is in direct relationship with upstream genetic susceptibility variants.

### IBD and epithelial and immune cell fractions of the coeliac duodenum share DMPs

As the IBD methylome is both, related to inflammation and genetic susceptibility, it may also be largely unspecific. We therefore chose coeliac disease (CeD), a chronic inflammatory condition of the GI tract with a well-characterized genetic component, to get further insight into methylome specificity. In addition, DNA methylation data for epithelial and immune components of CeD were analysed separately [34]. When we crossed IBD-DMPs with epithelial CeD-DMPs we found that, out of 4205 IBD-DMPs and 43 CeD epithelial-DMPs, 8 were common (representation factor = 17.7, p < 1.5e-08) (Table 6). Interestingly, 5/8 common DMPs mapped to the HLA region on chromosome 6. On the other hand, 31 IBD-DMPs were common with the 310 CeD immune-DMPs (representation factor = 9.5, p < 1e-20). These common hits were enriched for TGF-β signalling pathway (WikiPathways, adjusted p value = 0.04419), and were spread across the genome. All common DMPs followed the same direction (i.e., hypo or hypermethylation) in both diseases, indicating that methylation alterations were concordant. However, methylation fold changes were larger in CeD, probably due to the fact that the coeliac DMPs were identified in separated cell populations, while IBD methylation was assessed in whole intestinal tissue potentially blurring cell-specific signatures.

**Table 6.** IBD DMPs previously identified to be differentially methylated in both CeD duodenal epithelia and immune fractions.

CpG	Nearest gene	Mean Control	Mean IBD	Delta Beta	FDR IBD	FDR CeD
Epithelial fraction						
cg00403478	HLA-DPA1	6.2E-01	4.6E-01	−0.30	3.9E-04	7.51E-03
cg00676801	STAT1	7.8E-01	6.7E-01	−0.38	9.7E-05	1.40E-02
cg02181920	TAP1	5.2E-01	4.0E-01	−0.22	2.2E-06	1.65E-02
cg02286081	HLA-DPB1	7.9E-01	6.6E-01	−0.27	1.2E-02	1.84E-02
cg06471536	PREP	5.5E-01	4.4E-01	−0.17	1.4E-09	3.76E-02
cg07839457	NLRC5	5.5E-01	3.3E-01	−0.25	6.6E-12	1.71E-02
cg08735211	HLA-DMA	7.5E-01	5.9E-01	−0.31	2.8E-06	3.24E-02
cg09321817	HLA-DPA1	7.4E-01	4.8E-01	−0.38	7.3E-07	7.51E-03
Immune fraction						
cg01829342	FRMD6	4.4E-01	5.5E-01	0.19	1.3E-03	3.45E-02
cg01971120	RNU5 F-1	2.5E-01	3.7E-01	0.20	1.1E-11	1.93E-02
cg01977473	GRAMD2B	3.0E-01	4.1E-01	0.12	6.3E-13	3.74E-02
cg02360367	SKI	3.5E-01	4.8E-01	0.11	1.3E-11	9.49E-03
cg02909176	ATP11A	3.3E-01	4.5E-01	0.13	1.0E-10	1.07E-02
cg05364072	MAP7D1	5.2E-01	6.3E-01	0.22	5.0E-06	1.86E-02
cg07843390	GNG7	2.9E-01	4.0E-01	0.16	1.5E-12	4.04E-02
cg09558069	S100A5	3.4E-01	4.4E-01	0.13	3.7E-11	4.38E-02
cg09670127	BCL11A	3.3E-01	4.3E-01	0.13	3.0E-07	2.56E-02
cg09799714	PDZD3	2.4E-01	3.5E-01	0.15	2.4E-14	1.44E-02
cg10331073	APBB1IP	2.7E-01	3.9E-01	0.10	1.6E-07	4.32E-02
cg14997942	DNAJC1	3.3E-01	4.6E-01	0.23	1.1E-11	1.97E-02
cg15876825	VGLL4	3.9E-01	5.3E-01	0.11	6.9E-09	4.24E-02
cg15924102	LINC01132	2.9E-01	4.3E-01	0.13	5.2E-14	3.81E-02
cg16312609	MIR4708	2.8E-01	3.9E-01	0.17	1.1E-13	2.85E-02
cg17292100	SPAG1	2.5E-01	3.5E-01	0.12	1.3E-13	2.85E-02
cg17980364	TMEM135	2.8E-01	3.9E-01	0.12	1.2E-17	1.46E-02
cg18423737	SMAD7	5.6E-01	6.9E-01	0.13	8.6E-10	3.00E-02
cg18556822	NOSTRIN	2.4E-01	3.6E-01	0.18	1.7E-15	4.88E-03
cg19697512	PPCDC	3.9E-01	5.2E-01	0.15	7.7E-07	4.24E-02
cg19794481	MIR141	3.1E-01	4.3E-01	0.12	2.3E-12	3.18E-02
cg20059312	NGEF	3.7E-01	4.9E-01	0.10	1.1E-15	4.59E-02
cg20555562	TEX29	2.9E-01	4.2E-01	0.15	8.6E-08	2.79E-02
cg20859933	ZBTB44	3.4E-01	4.6E-01	0.13	1.6E-10	1.86E-02
cg21646082	CEP85	3.1E-01	4.2E-01	0.11	3.2E-11	4.77E-02
cg22601415	ANK3	3.6E-01	4.9E-01	0.11	7.6E-09	4.83E-02
cg22659049	LIMCH1	4.3E-01	5.9E-01	0.13	1.7E-03	4.24E-02
cg24216990	GUCA1A	2.5E-01	3.5E-01	0.15	1.4E-15	3.69E-02
cg26049390	RDH10	3.1E-01	4.2E-01	0.14	1.1E-04	2.16E-02
cg26075184	NKX2-3	2.2E-01	3.7E-01	0.15	5.4E-11	1.52E-02
cg26094842	VTI1A	3.0E-01	4.0E-01	0.19	1.1E-09	2.16E-02

In summary, there is a significant overlap in DNA methylation changes associated with IBD and CeD, including the HLA region.

## Discussion

IBD is a complex pathology with a wide range of clinical trajectories. Despite such heterogeneity, we show here that non-random changes in DNA methylation associated with IBD are robust to main clinical parameters and consistent across several studies.

There are intrinsic limitations of DNA methylation analyses relative to standard genetic profiling, such as confounding, reverse causation, and cellular heterogeneity [13,21]. Interpretability becomes even more complex when aggregating data from independent studies. Despite our efforts in limiting the effect of potential confounders, we are aware that the residual effect of cell composition, anatomical location, inflammation, etc., and/or the differences in sample size from the different studies may have influenced our results.

Different characteristics of DNA methylation, such as its relative stability, make this mark an ideal sensor of disease risk and progression. Indeed, several studies have been able to use DNA methylation as a marker of IBD in blood samples [31,35,36]. Both in blood and intestinal mucosa, a deeper mechanistic insight is necessary to better distinguish those methyl marks that are dependent on genetic susceptibility from those that are a consequence of environmental cues. We suggest here that IBD methylome is indeed a combination of both components, on the one hand, many associations at the site and region levels were enriched in inflammatory pathways, suggesting that methyl marks could have been introduced downstream of cytokine signalling (either up- or downs-stream of gene expression changes). On the other hand, at least a fraction of DNA methylation changes was linked to a neighbouring risk polymorphism, indicating an effector role for DNA methylation in the interface between genotype and phenotype.

In agreement with the largest study selected for our meta-analysis [10], genes near abnormal DNA methylation were enriched in immune and inflammatory pathways, highlighting the role of chronic inflammation in both, UC and CD. In particular, TGF-β is a cytokine able to modulate the inflammatory response, and it was enriched in IBD-DMRs. Moreover, it was enriched in those DMPs common between IBD and CeD, in agreement with the crucial role of TGF-β pathway in regulating the intestinal T cell response. An additional element that emerged from our pathway analysis is the potential crosstalk between IBD and adipogenesis. In fact, patients with IBD, particularly those with CD, develop ectopic adipose tissue (fat-wrapping or creeping-fat) covering a large part of the small and large intestine [37]. It has been proposed that in obese or overweight IBD patients it is the mesenteric adipose tissue that contributes to intestinal and systemic inflammation [37].

In our study, we identified 4532 CpG sites that simultaneously display differential variation and differential methylation (DVMCs) associated with IBD. In most cases, IBD mucosal cells displayed higher variation at those DVMCs relative to control cells. Although this hypervariability may represent cellular variation (e.g., changes in inflammatory or stromal components of the intestinal mucosa), it has been suggested that a stochastic component of methylation variation at certain genomic locations may characterize pathological conditions [28,38]. Of note, differential variation in DNA methylation has been found in other pathologies, including cancer [38–40]. In particular, they have been described as predictors of cancer development in non-tumour tissues [28,39] or associated with exposure to known carcinogens [41]. This is an interesting finding, considering that one fraction of IBD patients has an increased susceptibility to develop colon cancer [42].

In terms of genomic distribution, we found that DMPs are relatively absent from CGIs. Instead, they could be associated with other regulatory regions such as enhancers, for example, in association with SNPs. Indeed, GWAS performed in multiple complex diseases have shown that SNPs of susceptibility are enriched in enhancer regions, and DNA methylation could be an intermediary in this process [43,44]. Illustrating this, the presence of differentially methylated sites in the vicinity of known susceptibility loci supports the notion of DNA methylation as an intermediary between genotype and phenotype (mQTLs). In addition, among DMRs with a significant genetic association, we find *JAK3, KRT8, HLA* genes, all of them associated with a role in IBD pathogenesis [45–49].

The presence of CpGs participating in both IBD-DMPs as well as mQTLs suggests that a considerable number of the DMPs identified in our metanalysis are regulated by SNP-genotypes in cis. However, very few of these are associated with IBD. This observation points to the possibility that, although fine-mapping aims to identify the SNPs responsible for the disease-association, other nearby SNPs in strong linkage disequilibrium could be the ones implicated in the mQTLs, drawing the methylation patterns reported. Additionally, we describe a picture in which most of the IBD-DMPs seem to be genotype-independent, since they do not participate in any mQTL, at least in blood. Regarding the SNPs associated to IBD as well as to the methylation levels of IBD-DMPs, it is interesting that the methylation of a CpG island 4 kb upstream of the cg24032190-DMP identified in the first intron of *SMAD3* has been reported to be allele-specific and to regulate the expression of the gene [50]. Therefore, we propose another DMP in the same region that could mediate the association between the locus and IBD; and hypothesize that this could also be the case for the genomic regions surrounding *ADAM15, RNF186,* and *IRF5*.

Regarding coeliac epithelial DMPs also found altered in IBD, it is important to note that most of them were located in the HLA region. This locus presents strong linkage disequilibrium and encodes a number of genes related to immune response and immune regulation through self-recognition [49,51], and strongly predisposes to autoimmune diseases such as CeD. In our previous work [34], we claimed to have found a genotype-independent methylation signature in coeliac duodenal epithelia. The finding of a signature in the HLA region common to IBD and CeD reinforces this idea, given that the HLA association with IBD is much weaker (variance explained <5%) than with CeD, and moreover, different HLA haplotypes drive these associations [45]. Additionally, this common methylation signature points to a non-specific pattern, probably responding to common inflammatory forces in the two disorders.

## Conclusions

Our findings illustrate an aberrant DNA methylation landscape in IBD, independent of IBD subtype and other clinical and pathological features. The enrichment of abnormal DNA methylation in inflammatory pathways and genes suggests a direct role for this mark downstream of cytokine signalling and/or a risk genotype. Such a landscape may be a more general indicator of intestinal chronic inflammation, although evidence from purified epithelial cells suggests that those changes are not primarily explained by an inflammatory status [10]. Such effect of inflammation, as well as cell heterogeneity in general could not be directly accounted for in our analyses. However, we expect that such limitation will be compensated with the future addition of new IBD datasets with adequate and complete annotations. In addition, technological progress in other forms of methylation (e.g., 5hmC) and a higher coverage of the genome will add to the overall goal of identifying biomarkers in IBD.

## Methods

### Dataset selection

Dataset selection criteria included: methylome data obtained from intestinal mucosa (including colon and terminal ileum), availability of healthy controls and IBD samples (CD, UC, or both), in data obtained using Human Infinium Bead Arrays (Illumina’s HM450 or EPIC arrays), an established technology to detect DNA methylation [52]. Tables 1 & 2 shows the main characteristics of the datasets fulfiling these criteria. Dataset MTAB_3703/3709 was eventually excluded from the analyses as only 6 samples were of non-foetal origin, with only 3 samples from large intestine.

### Data preprocessing

All methylation data and sample information were downloaded from Gene Expression Omnibus (GEO) and Array Express public repositories, and analysed using R/Bioconductor packages [53]. Normalized data was loaded into R directly from each repository, except when raw idat files were also available. In that case, idat files were normalized using the “Funnorm“ function of the minfi package [54]. Each dataset was independently assessed for data quality and distribution, before merging. Merged data was filtered for sex chromosomes, known cross-reactive probes [55], and probes associated with common SNPs that may reflect underlying polymorphisms rather than methylation profiles [56]. In addition, the ‘nmode.mc’ function of the ENmIx package was used for the identification of multimodal sites [57]. These sites were not removed at this step but were used instead to classify significant associations in a later step.

### Quality control and cross-validation

After filtering, 392810 CpG sites common to all datasets were used to identify principal components (PC) of variation and plotted using PC regression and multidimensional scaling (MDS) plots. Strong associations were observed between PCs and known variables (i.e., dataset, sex, age, and anatomical location), with age and anatomical location partially confounded by the dataset of origin. As additional quality control, DNA methylation values were used to predict age and sex and contrast with downloaded phenotype information (Figure S1). Sex was inferred from the median total intensity signal on XY chromosomes and permitted the identification of eight sex mismatches that were removed from the analysis. Age prediction was performed using Horvath’s coefficients [58], as implemented in the wateRmelon package [59]. There was a strong positive correlation between reported and predicted age (Figure S1). For two datasets where age was not available, predicted age corresponded to adult samples, as reported in the corresponding repositories. The common merged and filtered matrix of methylation beta values and their corresponding phenotype data was taken to the next step.

As validation of our aggregated analysis, we performed independent region-level analyses to test for the association between IBD and DNA methylation in three datasets, where enough power made it possible (dataset 1: all datasets with available idat files, 2: dataset based on EPIC bead array data, and 3: dataset GSE42921). There was a significant overlap among those three analyses, with 905 common gene symbols (Figure S2). We also performed a leave-one-out cross-validation approach. To this end, we successively removed each of the six datasets of the study and performed differential methylation analysis at the probe and region levels (Figure S2). Two different diagrams are shown due to limitations of this visualization, but they illustrate that there is a common set of CpG sites differentially methylated across all or most datasets, and an important overlap with our final list of differentially methylated probes. Similar results were obtained when differential methylation was studied at the region level (DMRs).

### Latent variables and batch correction

In addition to the obvious batch effect of the dataset of origin, DNA methylation is known to be influenced by genotype, sex, age, and cell composition. As all of these factors are potential confounders, we tried to minimize or account for their effect using different strategies. Those factors where data was available (i.e., dataset, sex, predicted age) were modelled in a linear regression. In the particular case of sex where the effect on DNA methylation is strong, we removed an important part of such effect by filtering out all probes mapping to chromosomes X and Y, as described above. The effect of genotype was addressed a posteriori, in our mQTL analyses. For all other factors (except inflammation, where annotated data was not available for most samples), we were able to assess their association with the main components of variation before and after adjustment for latent variables identified using surrogate variable analysis (SVA) [60]. In particular, cell composition has been shown to be suited to be addressed using this strategy [61]. In our case, cell composition can be dependent on both, inflammation and anatomical location. Anatomical location was indeed strongly associated with the first component of variation (PC1) (Figure S2), an effect that was attenuated after SVA. A similar reduction in the strength of association with main PCs was observed for the effect of dataset, age, and sex. Of note, our variable of interest (IBD vs. control) was associated with the first three PCs after SVA adjustment, while the effect of all other co-variates and batches was minimized (Figure S1). In total, 29 surrogate variables were identified and they were modelled in our linear regression, together with dataset, sex, and age. There was no association (using linear regression) between surrogate variables (SVs, Figure S2) and our main variable. However, dataset of origin and anatomical location were strongly associated with several SVs (Figure S2).

### Differential methylation

Associations were tested for 392810 CpG sites, across 285 samples (81 control and 204 IBD samples). Methylation data was modelled at the probe and region levels using a linear model with Bayesian adjustment [62]. Sex and dataset were modelled together with subject status (i.e., control or IBD patient). Surrogate variables identified in the previous step were also included in the linear model to account for unknown sources of variation. Quantile-quantile (QQ) plots were used to inspect the distribution of resulting p values and estimate statistical inflation (Figure S2). Differentially methylated positions (DMPs) and regions (DMRs) were selected based on a methylation change (delta beta) of at least 10% or 5% (for DMPs and DMRs, respectively) when comparing control vs. IBD samples and a false discovery rate – (FDR) adjusted p value below 0.05. DMRs were identified with the DMRcate package using the recommended proximity-based criteria [63]. A DMR was defined by the presence of at least two differentially methylated CpG sites with a maximum gap of 1000 bp. To identify CpG positions exhibiting significant differential variation and differential methylation (DVMCs), data was analysed using iEVORA, an algorithm that identifies DNA methylation outlier events shown to be indicative of malignancy [28]. iEVORA is based on Bartlett’s test (BT) that examines the differential variance in DNA methylation, but because BT is very sensitive to single outliers, it is complemented with re-ranking of significant events according to t-statistic (TT, t test), to balance the procedure. The significance is thus assessed at the level of differential variability, but the significance of differential variability with larger changes in the average DNA methylation are favoured over those with smaller shifts. We used adjusted q(BT) <0.001 and p(TT) <0.05 as thresholds for significant DVMCs. To study genomic context, we used HM450 annotations, with hg19 as the human reference genome, UCSC and previously reported genomic features [65]. Differentially methylated genes (DMPs, DMRs, and DVMCs) were further analysed to determine functional pathways and ontology enrichment using Enrichr [56]. We tested the association between two gene lists by calculating a hypergeometric distribution using the ‘phyper’ function implemented in R base. To this end, we used the gene list lengths, their overlap, and a conservative total number of sites (400 k for data based on HM450 bead arrays). Based on the same distribution, we calculated the random expectation and the corresponding proportion between the observed overlap and such expectation. This value is referred to as ‘representation factor’ throughout the text.

### SNPs-DMPs associations in IBD and CeD

To identify methylation quantitative trait loci (mQTL), single nucleotide polymorphisms (SNPs) associated with IBD risk were obtained from a fine-mapping study of IBD with single-variant resolution [29]. Two independent GWAS were also considered in some of the analyses: (1). Jostins L et al. [32], and (2). Lange KM de et al. [30]. Genomic distances between 368 unique SNPs pooled from these three studies and IBD-associated DMPs were calculated using the R package GenomicRanges. In addition, we searched for those CpGs that apart from being differentially methylated in IBD according to our metanalysis, were previously reported to be differentially methylated in a previous work performed by our group in CeD [34]. CeD is a genetic, inflammatory condition of the duodenum in which the Human Leucocyte Antigen (HLA) region explains around 40% of the heritability, and HLA-DQ2/-DQ8 molecules are necessary for gliadin presentation and activation of the autoimmune response. Briefly, we looked for the overlap between the bimodal IBD-DMP list presented here and the coeliac DMPs found in both the epithelial and the immune cell fractions of the duodenum. We also searched for the IBD-DMPs that were previously reported to participate in blood mQTLs in cis (2 Mb, p < 1e-6), according to the largest to-date mQTL database available [33], and found the overlap between them and the SNPs associated to IBD [29]. All the overlaps were reported using in-house R scripts. We also calculated the representation factor and the associated probability of the overlaps (hypergeometric test), in order to establish whether they were significant.

## Supplementary Material

Supplemental Material

## Data Availability

Demographic, clinical, and genomic data used in the present study have been published in open access repositories and are available to the public (Table 1). No additional datasets were generated or analyzed during the current study.

## Preprint

This manuscript can be accessed as a preprint version at the following link:

https://www.biorxiv.org/content/10.1101/565200v1
