# Supplementary material for: The DNA methylome of inflammatory bowel disease (IBD) reflects intrinsic and extrinsic factors in intestinal mucosal cells
Source: Epigenetics. 2020 Apr 12;15(10):1068–82. doi: 10.1080/15592294.2020.1748916 (PMC7518701; doi:10.1080/15592294.2020.1748916)
Supplement: Supplemental Material [file KEPI_A_1748916_SM5561.zip › Supplementary caption.docx]

**Supplementary Data**

**Table S1.** Full list of differentially methylated positions (DMPs).

**Table S2.** Full list of differentially methylated regions (DMRs).

**Table S3.** Full list of differentially variable and methylated CpGs (DVMCs).

**Table S4.** Overlap between bimodal IBD-DMPs and CpGs participating to blood mQTLs as defined by McRae et al. ^33^.

**Figure S1.** Data quality and preprocessing. **A.** Sex predictions using XY chromosome methylation data. **B.** Predicted (upper left panel) and reported (upper right panel) age. Reported age was not available for the two datasets that studied adult subjects. Matching between reported and predicted age in control and IBD samples (lower left and right panels, respectively). **C.** Multidimensional scaling (MDS) plots, according to bead array version (EPIC vs HM450), dataset, sex, condition (control vs IBD), anatomical location (asc_col: ascending colon, sig_col: sigmoid colon, ter_ile: terminal ileum, ND: no data available), and IBD subtype (CD vs UC). **D.** Principal component regression analysis (see Methods) before (left) and after (right) adjustment.

**Figure S2.** Latent variables and cross-validation. Surrogate variable analysis was perfomed, as described in Methods. The plots show the association between the three main latent variables (SV1, SV2, and SV3) and our variable of interest, which distinguishes IBD from healthy tissues (**A)**. A similar plot was done for sex (**B**), study dataset (**C**), and anatomical location (**D**). **E.** Quantile-quantile plot for the association between DNA methylation at the probe-level and sample type (IBD vs control). Statistical inflation (λ) is indicated on the plot. **F.** Independent DMR analysis (IBD vs control) was performed in 3 of our 6 datasets, where enough power made it possible (HM450: all datasets with available idat files, EPIC: dataset based on EPIC bead arary data, and dataset GSE42921). The venn diagram shows the significant overlap among those 3 analyses, with 905 common gene symbols. **G**. Leave-one-out cross-validation was done by alternatively removing each of the six datasets of the study and performing differential methylation analysis at the probe level. Each dataset is named as out1, out2, etc, after the removed dataset. The left panel shows the overlap between the final list of DMPs and a subset of the analyses (out1, out2, out3, and out6). An addition venn diagram is shown on the right panel with another subset of the analyses (out2, out3, out4, out5, and out6), due to limitations of this visualization.

**Figure S3.** Differential methylation analysis. Distribution of the top DMPs across the different datasets (compare with Fig 1A). Top hypo- (**A**) and hyper-methylated (**B**) DMPs are shown. Barplots are shown for control (gray) and IBD (red) samples separately for each dataset. **C.** Genomic distances were calculated between all DMPs identified and known SNPs associated with IBD risk, according to three independent studies ^29,30,32^. Top violin plot shows the distribution of the distances using the aggregated data from the three studies (red). The remaining three plots show the distances independently for each of the studies. All: all DMPs, Hypo: DMPs hypomethylated in IBD, Hyper: DMPs hypermethylated in IBD, HM450: all informative CpG sites in the Infinium bead array.
