# Supplementary figures and images for: The DNA methylome of inflammatory bowel disease (IBD) reflects intrinsic and extrinsic factors in intestinal mucosal cells
Source: Epigenetics. 2020 Apr 12;15(10):1068–82. doi: 10.1080/15592294.2020.1748916 (PMC7518701; doi:10.1080/15592294.2020.1748916)

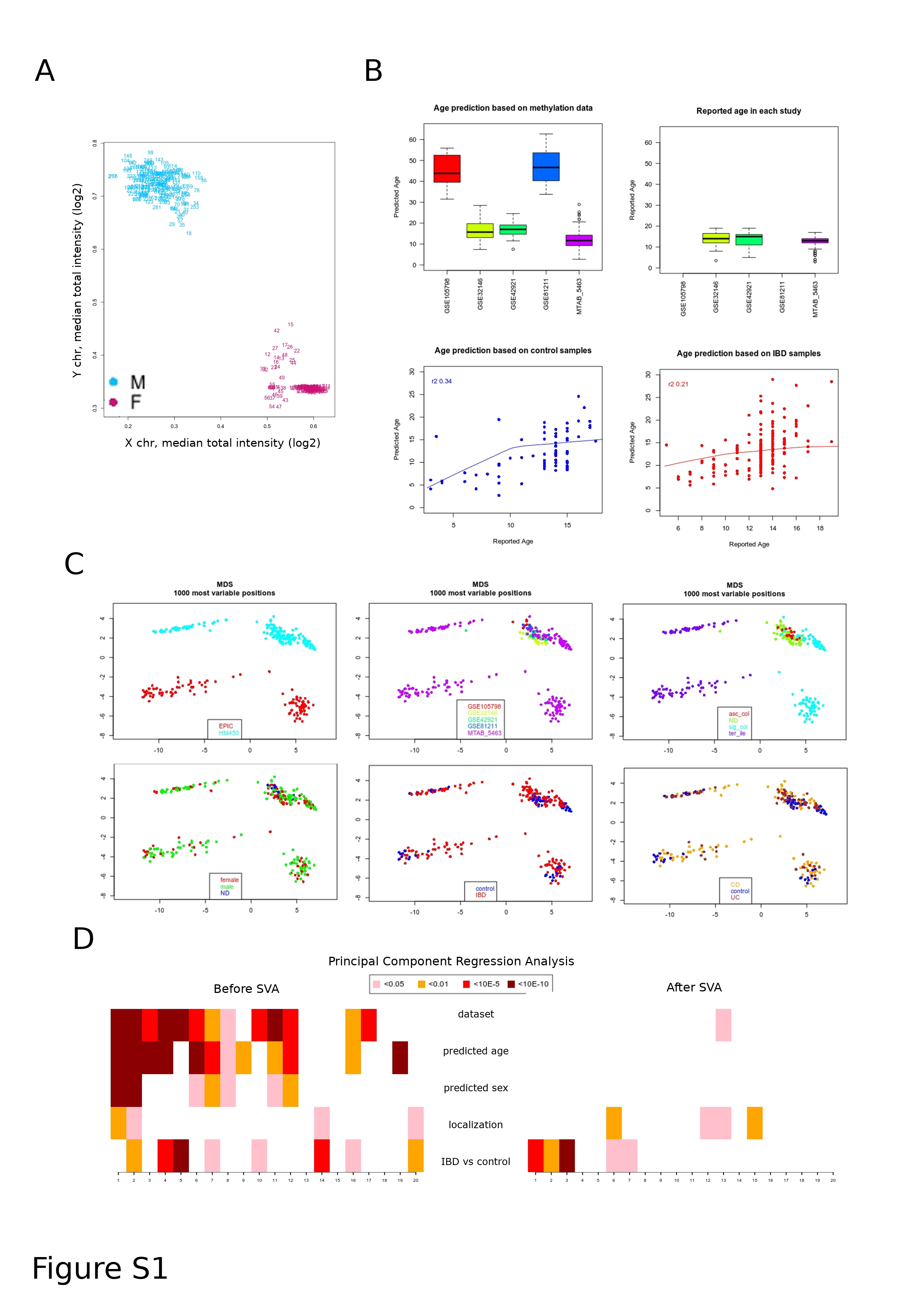

Supplement: Supplemental Material [file KEPI_A_1748916_SM5561.zip › FigS1.tif]

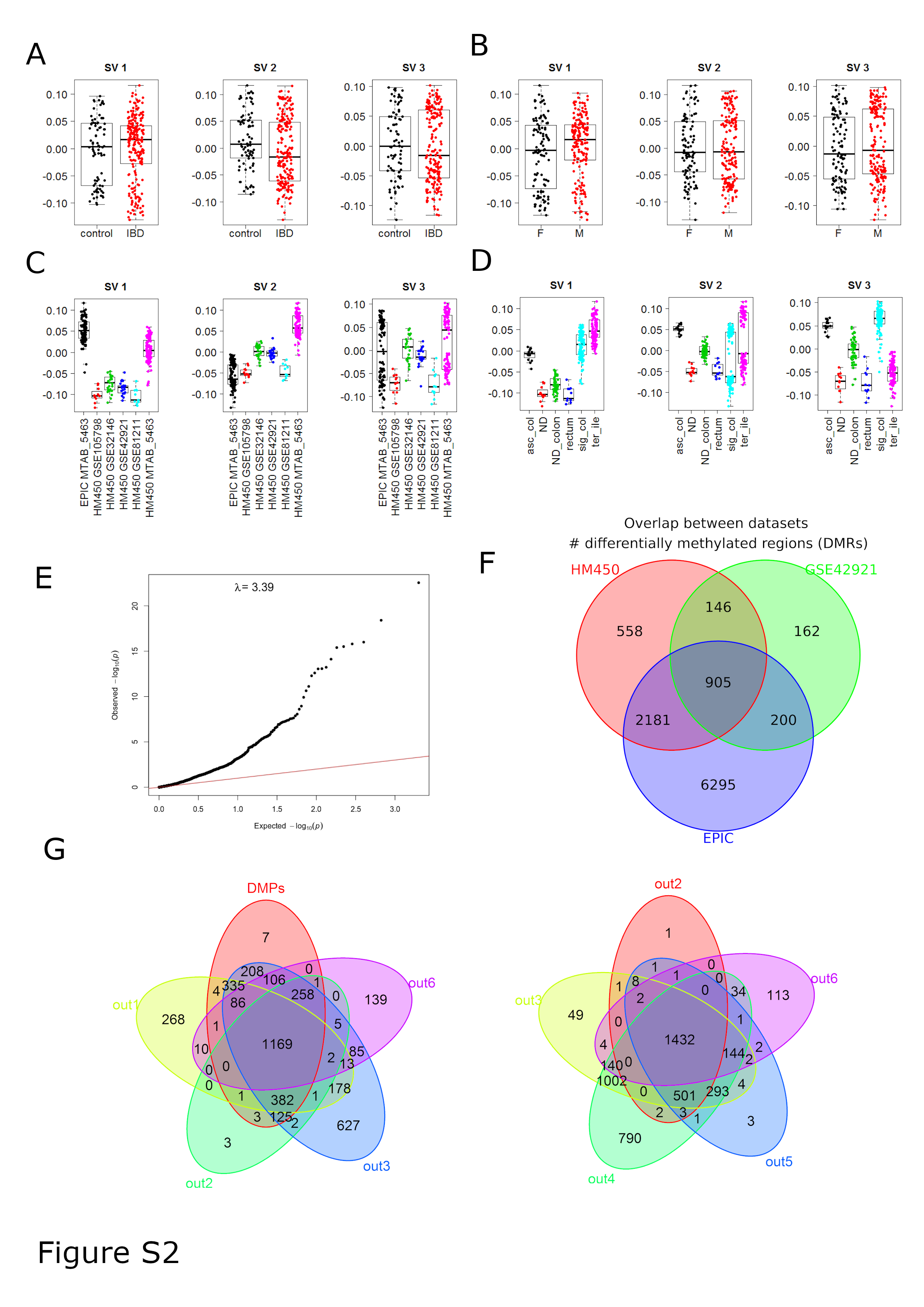

Supplement: Supplemental Material [file KEPI_A_1748916_SM5561.zip › FigS2.tif]

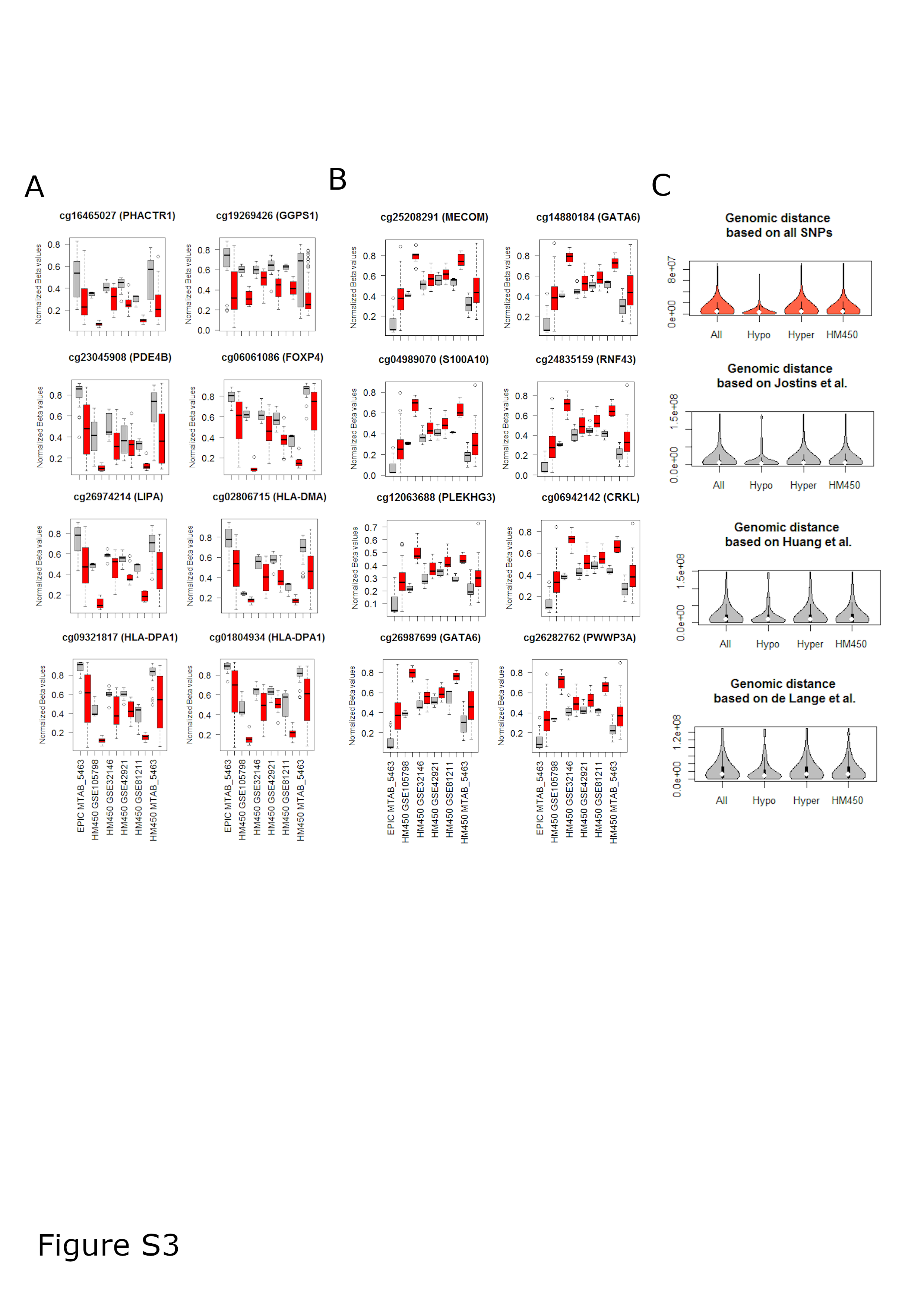

Supplement: Supplemental Material [file KEPI_A_1748916_SM5561.zip › FigS3.tif]
